# 

**Published:** 1896-03

**Authors:** 


### CONTENTS.

COMMUNICATIONS.

Chairman's Address ..... 105
Phisiological Antagonism between Medicines and Diseases ..... 113
Dentistry a Distinct and Indeı endent Profession ..... 116
The Originator of the Quinine Industry ..... 120
Shadowgraphs without the Crooke's Tubes ..... 121

SELECTIONS.

Shun Bolted Flour—Dr. John Ellis tells why Americans Lose their Teeth at an Early Age ..... 124
Child Study ..... 127
Effectual Treatment of Acute Alcoholism ..... 129
Solutions ..... 130
The Best Antiseptic ..... 130
Alcohol and Happiness ..... 131
Surgical Uses of Cocaine ..... 132
Comparative Vitality of the Sexes ..... 133
Potassium Iodide ..... 133
Anything to Get Well ..... 134
Professor Gray ..... 135
Fate of a Famous Health Resort ..... 136
Ill-Temper a Symptom of Excessive Meat-Eating ..... 136
The Prevention of Consumption ..... 137
Biting the Nails ..... 138
Pregnancy and Dental Caries ..... 138
Unprofessional Conduct ..... 139
Harvard Medical Faculty ..... 139
The Risks of Anesthesia ..... 140
Arsenical Poisoning ..... 140
Elimination Through the Stomach ..... 141
Neuralgia ..... 142
Liquid Air ..... 142
Separation of Teeth ..... 143
Childrens's Food ..... 143
The Triumphs of Electricity ..... 144
To Estimate the Dampness of a House ..... 144
The Secret of Centenarianism ..... 145
Surgery Without Pain ..... 147
The Use and Abuse of the Brain ..... 149
Washington City Dental Society ..... 150
Early Rising and Insanity ..... 150

EDITORIAL.

Ohio State Dental Law ..... 152
The Medical Law ..... 152
Mississippi Valley Dental Association ..... 156

OBITUARY.

Dr. Harvey Scott ..... 153

Dr. W. H. Dwinellf ..... 154

H. R. Bingham ..... 155

